# High incidence of inguinal hernias among patients with congenital abdominal wall defects: a population-based case–control study

**DOI:** 10.1007/s00431-021-04172-2

**Published:** 2021-06-25

**Authors:** Arimatias Raitio, Nelly Kalliokoski, Johanna Syvänen, Samuli Harju, Asta Tauriainen, Anna Hyvärinen, Mika Gissler, Ilkka Helenius, Ulla Sankilampi

**Affiliations:** 1 Department of Paediatric Surgery, University of Turku and Turku University Hospital, Turku, Finland; 2 Department of Surgery, Kainuu Central Hospital, Kajaani, Finland; 3 Department of Paediatric Surgery, University of Tampere and Tampere University Hospital, Tampere, Finland; 4 Information Services Department, Finnish Institute for Health and Welfare, Helsinki, Finland; 5 Department of Neurobiology, Care Sciences and Society, Karolinska Institute, Solna, Sweden; 6 Department of Orthopaedics and Traumatology, University of Helsinki and Helsinki University Hospital, Helsinki, Finland; 7 Department of Paediatrics, Kuopio University Hospital, Kuopio, Finland

**Keywords:** Exomphalos, Gastroschisis, Inguinal hernia, Omphalocele

## Abstract

The aim of this nationwide population-based case–control study was to assess the incidence of inguinal hernia (IH) among patients with congenital abdominal wall defects. All infants born with congenital abdominal wall defects between Jan 1, 1998, and Dec 31, 2014, were identified in the Finnish Register of Congenital Malformations. Six controls matched for gestational age, sex, and year of birth were selected for each case in the Medical Birth Register. The Finnish Hospital Discharge Register was searched for relevant diagnosis codes for IH, and hernia incidence was compared between cases and controls. We identified 178 infants with gastroschisis and 150 with omphalocele and selected randomly 1968 matched, healthy controls for comparison. Incidence of IH was significantly higher in gastroschisis girls than in matched controls, relative risk (RR) 7.20 (95% confidence interval [CI] 2.25–23.07). In boys with gastroschisis, no statistically significant difference was observed, RR 1.60 (95% CI 0.75–3.38). Omphalocele was associated with higher risk of IH compared to matched controls, RR 6.46 (95% CI 3.90–10.71), and the risk was equally elevated in male and female patients.

*Conclusion*: Risk of IH is significantly higher among patients with congenital abdominal wall defects than in healthy controls supporting hypothesis that elevated intra-abdominal pressure could prevent natural closure of processus vaginalis. Parents should be informed of this elevated hernia risk to avoid delays in seeking care. We also recommend careful follow-up during the first months of life as most of these hernias are diagnosed early in life.

**Table:** 

**What is Known:** *• Inguinal hernia is one of the most common disorders encountered by a pediatric surgeon.* *• Prematurity increases the risk of inguinal hernia.*	
**What is New:** *• Children with congenital abdominal wall defects have a significantly higher risk of inguinal hernia than general population.* *• Families should be informed of this elevated hernia risk to avoid delays in seeking care.*

**What is Known:**

*• Inguinal hernia is one of the most common disorders encountered by a pediatric surgeon.*

*• Prematurity increases the risk of inguinal hernia.*

**What is New:**

*• Children with congenital abdominal wall defects have a significantly higher risk of inguinal hernia than general population.*

*• Families should be informed of this elevated hernia risk to avoid delays in seeking care.*

## Introduction

Inguinal hernia (IH) is one of the most common disorders encountered by a pediatric surgeon with an estimated incidence between 8 to 50 of every 1000 live births in general population and rising up to 20% in extremely premature infants [1, 2]. Also, an inverse correlation of birth weight and incidence of IH has been observed [3, 4]. Male predominance in IH has been reported in several studies [1, 2, 5], and it has been speculated to be related to testicular descent and/or incomplete obliteration of processus vaginalis [4, 6]. Other reported risk factors for IH include chronic lung disorders, need for mechanical ventilation, especially use of high-frequency oscillating ventilator, and postnatal dexamethasone exposure [2, 4, 7]. Furthermore, elevated risk of IH has been reported after ventriculo-peritoneal shunt operation [8–10]. It has been speculated that increased intra-abdominal pressure caused by mechanical ventilation or shunt operation would predispose these patients to IH [4, 9].

Gastroschisis and omphalocele are relatively rare congenital anomalies with respective live birth prevalences of 1.73 and 1.69 per 10,000 in Finland [11, 12]. Gastroschisis often presents as an isolated anomaly [11, 13], whereas omphalocele is often associated with other severe comorbidities including chromosomal abnormalities and cardiac defects [12, 14, 15]. At the time of closure of the abdominal wall defect, these infants are often exposed to significantly elevated intra-abdominal pressures requiring continuous monitoring [16–18].

There is substantial evidence that abdominal wall defects are often associated with undescended testicles [19–21], and a high frequency of groin surgery has also been observed in these patients [22]. Furthermore, a high frequency of IH has been reported earlier among omphalocele patients [23, 24]. However, these findings are based on a rather small number of patients and both studies included giant omphalocele cases only. Therefore, we set out to explore this potential association in a population-based setting. We hypothesized that children with abdominal wall defects would have a higher incidence of IH than general population.

## Material and methods

The study is based on the records of the Finnish Register of Congenital Malformations (FRM), the Medical Birth Register (MBR), and the Finnish Hospital Discharge Register (FHDR), all maintained by the Finnish Institute for Health and Welfare (THL). The diagnoses in FRM are coded according to an extended version of the 9th Revision of the International Classification of Diseases (ICD-9) of the World Health Organization and according to ICD-10 in FHDR. Nationwide data on all inpatient hospital discharges and outpatient visits in public hospitals are registered in FHDR. The data quality and coverage of these registers has been validated and considered good in several studies [25–28]. The study population identified in FRM was cross-linked with the FHDR data by a unique personal identification code.

### Selection of cases and controls

All gastroschisis and omphalocele cases born between Jan 1, 1998, and Dec 31, 2014, were identified in the FRM. For every patient with gastroschisis or omphalocele, six controls without major structural anomalies and chromosomal defects were selected from the MBR. The controls were matched for gestational age (± 1 week), sex, and year of birth.

### Outcome measures and data collection

Primary outcome measure was diagnosis of IH. The incidence of IH was compared between gastroschisis and omphalocele cases and their matched controls. Secondary outcome measure was age at diagnosis, which was calculated for the first recorded diagnosis of IH in each patient. Hospital admissions and outpatient visits were analyzed between Jan 1, 1998, and Dec 31, 2015, allowing a minimum 1-year follow-up. FHDR was searched for relevant ICD-10 codes for IH (K40). Unilateral and bilateral hernias were recorded separately.

### Statistical analysis

Chi-square and Fisher’s exact tests were utilized to analyze categorical variables. A significance level of p < 0.05 (two-tailed) was set. Relative risks (RR) with 95% confidence interval (CI) for incidence of IH were calculated. One-way Anova and Wilcoxon rank test were used to compare continuous variables. Analyses were performed using JMP Pro, version 15.1.0 for Windows (SAS Institute Inc., Cary, North Carolina, USA).

### Ethical considerations

The approval of the Institutional Review board at Turku University Hospital was obtained before conducting this study. The Finnish Institute for Health and Welfare gave a permission to use their health register data in this study.

## Results

We identified 178 infants with gastroschisis and 150 with omphalocele in the registers born between Jan 1, 1998, and Dec 31, 2014.

### Gastroschisis

Ninety-seven (54%) of the gastroschisis patients were born prematurely before 37 gestational weeks. With a median follow-up time of 8.2 years (range 1.0–18.0), 14 (7.4%) patients with gastroschisis were diagnosed with IH including 6/86 (7.0%) girls and 8/92 (8.7%) boys (Table 1). Cryptorchidism was operated on 2/8 (25%) boys with IH. Only 5/516 (1.0%) girls and 30/552 (5.4%) boys among matched controls had a diagnosis of IH. Two gastroschisis patients (1 female, 1 male) and four controls (1 female, 3 males) had bilateral IH with no significant difference between groups (*P* = 0.18). There was no statistically significant difference in patients’ age at the time of diagnosis between cases and controls, *P* = 0.10.

**Table 1:** Incidence of inguinal hernia among female patients with gastroschisis was significantly higher than their matched controls. Values given as median and range

	All			Females			Males		
	Gastroschisis, *n* = 178	Control, *n *= 1068	*P* value	Gastroschisis, *n* = 86 (48%)	Control,* n* = 516 (48%)	*P* value	Gastroschisis,* n* = 92 (52%)	Control, *n* = 552 (52%)	*P* value
Gestational age (weeks)	36.7, (27.6–40.3)	36.8, (26.6–40.3)	0.88	36.6, (29.0–40.1)	36.6, (28.4–40.1)	0.99	36.9, (27.6–40.3)	36.9, (26.6–40.3)	0.83
Age at diagnosis (years)	0.2 (0.0–4.8)	0.7, (0.1–5.4)	0.10	0.3 (0.1–0.6)	0.4 (0.2–2.1)	0.27	0.2 (0.0–4.8)	0.8 (0.1–5.4)	0.17
Inguinal hernia cases (%)	14 (7.9%)	35 (3.3%)	0.01	6 (7.0%)	5 (1.0%)	0.002	8 (8.7%)	30 (5.4%)	0.23
Relative risk (95% confidence interval)	2.40 (1.32–4.37)			7.20 (2.25–23.07)			1.60 (0.75–3.38)		

The risk of IH was significantly higher in gastroschisis than in matched controls, RR 2.40 (95% CI 1.32–4.37). When sexes were analyzed separately, girls with gastroschisis were significantly more likely to develop IH than their controls (RR 7.20, 95% CI 2.25–23.07). No statistically significant difference was observed in males, RR 1.60 (95% CI 0.75–3.38) (Table 1). Median gestational age was comparable in gastroschisis patients with and without IH, 36.5 weeks (range 34.0–40.3) vs 36.8 (27.6–40.1), respectively (*P* = 0.31). In the controls, prematurity was associated with significantly higher incidence of IH; median gestational age was 35.6 (26.6–38.9) among those with diagnosed IH and 36.9 (27.0–40.3) in those without hernia, *P* = 0.0002.

### Omphalocele

The median follow-up time for omphalocele cohort was 9.9 years (range 1.2–17.9). Only 41 (27%) of the omphalocele patients were born preterm before 37 gestational weeks. There were 3/67 (4.5%) female omphalocele patients with diagnosed IH (Table 2). The highest incidence of IH was observed among boys with omphalocele, 25/83 (30.1%). Only 3/402 (0.8%) of the girls and 23/498 (4.6%) boys in control group had a diagnosis of IH. There was no statistically significant difference in the age at diagnosis in either sex. Surgery on undescended testes was required in 7/25 (28%) boys with IH.

**Table 2:** Incidence of inguinal hernia among omphalocele patients was significantly higher than among their matched controls. Values given as median and range

	All			Females			Males		
	Omphalocele, *n* = 150	Control, *n* = 900	*P* value	Omphalocele, *n* = 67 (45%)	Control, *n* = 402 (45%)	*P* Value	Omphalocele, *n* = 83 (55%)	Control, *n* = 498 (55%)	*P* Value
Gestational age (weeks)	38.6 (23.4–42.3)	38.6 (23.4–42.3)	0.90	38.7 (23.4–41.3)	38.7 (23.4–41.3)	0.99	38.4 (27.4–42.3)	38.4 (26.6–42.3)	0.86
Age at diagnosis (years)	0.2 (0.1–8.3)	0.3 (0.0–7.2)	0.18	1.7 (0.3–4.8)	2.3 (0.5–4.6)	0.83	0.2 (0.1–8.3)	0.3 (0.0–7.3)	0.18
Inguinal hernia cases (%)	28 (18.7%)	26 (2.9%)	< 0.0001	3 (4.5%)	3 (0.8%)	0.04	25 (30.1%)	23 (4.6%)	< 0.0001
Relative Risk (95% Confidence Interval)	6.46 (3.90–10.71)			6.00 (1.24–29.11)			6.52 (3.89–10.93)		

Omphalocele was associated with an even higher risk of IH than gastroschisis compared to matched controls, RR 6.46 (95% CI 3.90–10.71). Both male and female patients with omphalocele were approximately six times more likely to develop IH than their matched controls (Table 2). Also, bilateral hernia was significantly more common in omphalocele patients: 8/150 (5.3%) vs 4/900 (0.4%), *P* < 0.0001. All bilateral hernias were observed in males. Lower gestational age was significantly associated with IH in omphalocele controls but among cases the difference was only borderline significant. Median gestational age for omphalocele patients with IH was 38.1 (27.4–41.9) vs 38.7 (23.4–42.3) in those without hernia, *P* = 0.05. The corresponding numbers for control group were 35.3 (24.4–40.6) with hernia and 38.6 (23.4–42.3) without hernia, *P* < 0.0001.

## Discussion

According to this population-based case–control study, the incidence of IH is significantly higher in patients with congenital abdominal wall defects than their matched controls. Omphalocele carries approximately sixfold risk of IH, while girls with gastroschisis are up to seven times more likely to develop IH. However, no elevated risk of IH was observed in male infants with gastroschisis.

Male predominance of IH patients is supported by all published reports. However, male-to-female ratio varies from two up to 12-fold risk in males [1, 2, 4, 5]. Interestingly, IH presented with almost equal gender distribution in gastroschisis patients (7.0% females vs 8.7% in males) with no statistical difference (*P* = 0.78). However, the other results reported here were in keeping with previous findings. Both omphalocele patients and control groups were dominated by males approximately 6:1. In our cohort, co-occurrence of cryptorchidism requiring surgery and IH was equally observed in gastroschisis (25%) and omphalocele (28%) with lacking data on laterality. Contrary to omphalocele, normal testicular descent is probable in gastroschisis patients with cryptorchidism at birth [20]. According to the current study, natural closure of processus vaginalis also appeared more likely in boys with gastroschisis than omphalocele. On the other hand, the risk of IH in girls with gastroschisis and omphalocele was equally elevated, suggesting that natural obliteration of processus vaginalis is disturbed in females with abdominal wall defects. However, the incidence of IH in boys with gastroschisis was not elevated, suggesting that other factors in addition to raised intra-abdominal pressure affect the closure of processus vaginalis.

Prematurity is associated with high incidence of IH, and the lower the birth weight, the higher the incidence of IH [3, 4]. This was also supported by our findings among control group, where gestational age was significantly lower in IH patients. In omphalocele patients, IH was associated lower gestational age, yet with only borderline statistical significance. However, in gastroschisis, gestational age was not associated with the risk of IH.

Almost all pediatric IHs are of indirect type [29] and caused by an incomplete closure of processus vaginalis, which is a prerequisite for the development of IH [10]. Studies on patients with ventriculo-peritoneal shunts have revealed a higher incidence of both hydrocele [10] and IH [8–10] among these patients. This has led to the hypothesis that raised intra-abdominal pressure or irritation caused by cerebrospinal fluid may prevent a natural closure of the processus vaginalis or alternatively, may convert a patent processus vaginalis to a clinical hernia [8, 10]. The findings of the current study reported up to sixfold incidence of IH in patients with congenital abdominal wall defects. As these patients are exposed to elevated intra-abdominal pressures in early neonatal period, our findings support the hypothesis of raised intra-abdominal pressure preventing natural closure of the processus vaginalis.

### Strengths and limitations

The strength of the current study is population-based data and six controls for each case with abdominal wall defects matched for the most relevant risk factors (i.e., sex and gestational age) for IH. Also, the register data stored in the FRM, the MBR, and the FHDR are all validated with high accuracy and a full country-coverage [27, 30–32]. All hospitals in our country report to these registers, and there are no private children’s hospitals in Finland. Furthermore, hospitals are expected to report the diagnosis and operation codes accurately as they are the basis for hospital billing [33]. The weakness of this study is the shorter follow-up time in cases born recently.

## Conclusions

In conclusion, patients with gastroschisis and especially those with omphalocele are significantly more likely than general population to develop IH. These findings support the hypothesis that elevated intra-abdominal pressure could prevent natural closure of processus vaginalis exposing these infants to development of IH. Due to high incidence of IH among these patients, we recommend appropriate parental guidance to avoid delays in seeking care.

## Data Availability

The data that support the findings of this study are available from the corresponding author upon reasonable request.

## Abbreviations

CI: Confidence interval
FHDR: Finnish Hospital Discharge Register
FRM: Finnish Register of Congenital Malformations
ICD: International Classification of Diseases
IH: Inguinal hernia
MBR: Medical Birth Register
RR: Relative risk
THL: Finnish Institute for Health and Welfare

## Code availability

JMP Pro, version 13.1.0 for Windows (SAS Institute Inc., Cary, North Carolina, USA).
